# Supplementary material for: Comprehensive identification of SWI/SNF complex subunits underpins deep eukaryotic ancestry and reveals new plant components
Source: Commun Biol. 2022 Jun 6;5:549. doi: 10.1038/s42003-022-03490-x (PMC9170682; doi:10.1038/s42003-022-03490-x)
Supplement: Supplementary file 2 — Supplementary Information [file 42003_2022_3490_MOESM2_ESM.pdf]

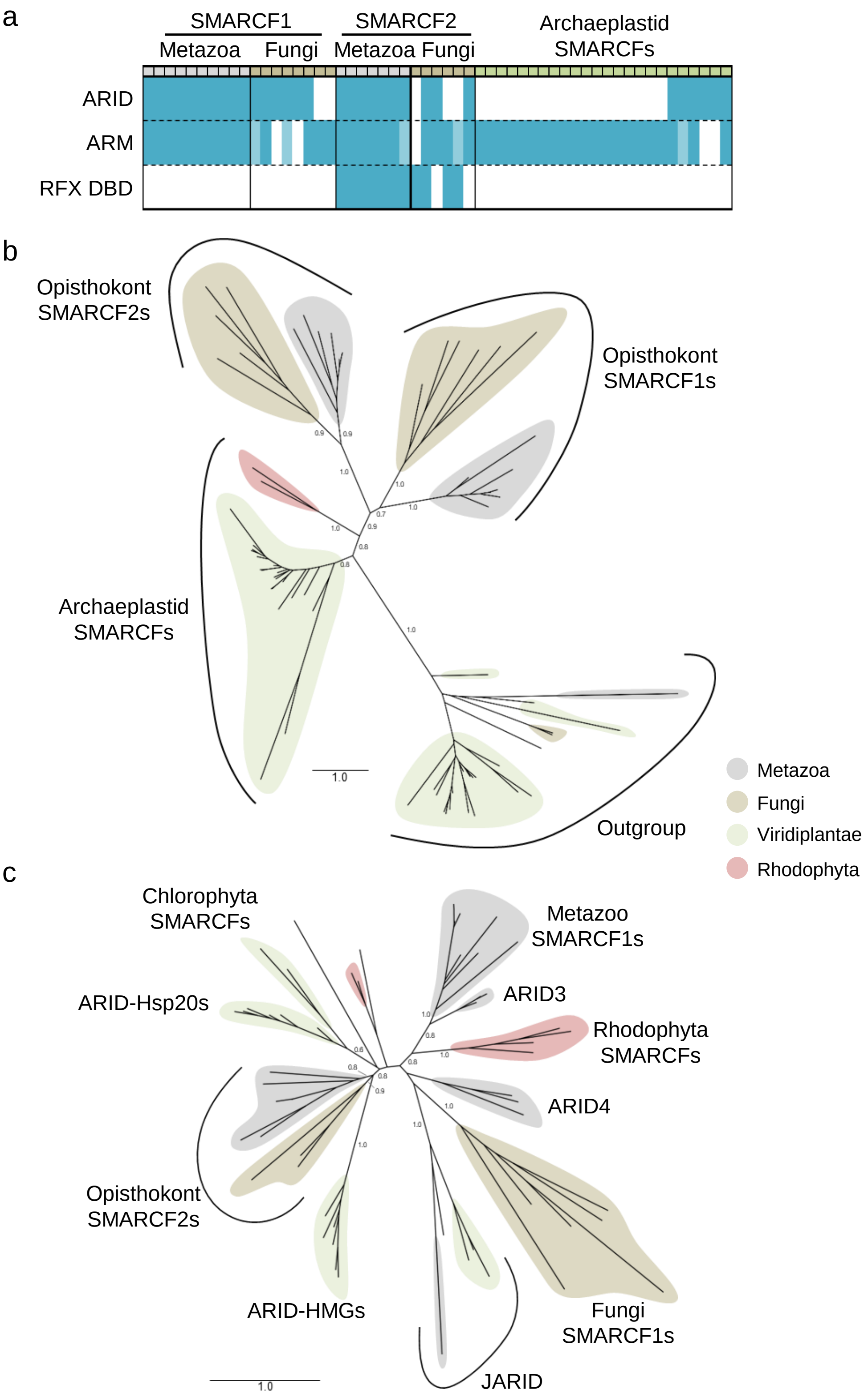

Supplementary Figure 1. SMARCF architectures have evolved around a conserved Armadillo fold. a) Occurrence of ARID, ARM-fold and RFX-DNA binding domain (DBD) in SMARCF proteins from different species. Columns represent independent species from the indicated lineages and assigned to SMARCF1/2 paralog groups in the case of ophistokonts. Blue indicates bona fide presence of a given domain (rows) as found by Pfam or InterProScan analyses. Light blue represents the presence of non-automatically findable domains that could be recovered by alignment with Pfam/InterProScan-detectable domains. Empty lines indicate complete absence of a given domain. b) Maximum likelihood phylogenetic analysis of ARM-folds from SMARCF proteins and related ARM-domains (outgroup). c) Maximum likelihood phylogenetic analysis of ARID domains from SMARCF proteins and related ARID-containing proteins. Support values associated with main branches represent maximum likelihood bootstrap values from 1000 replicates. Scale bar, ratio of substitutions per site. Raw phylogenetic tree files can be found at https://data.mendeley.com/datasets/6m4b8zrnpt/draft?a=d3801829-a6f1-4865-96b8-7d3138261e7b.

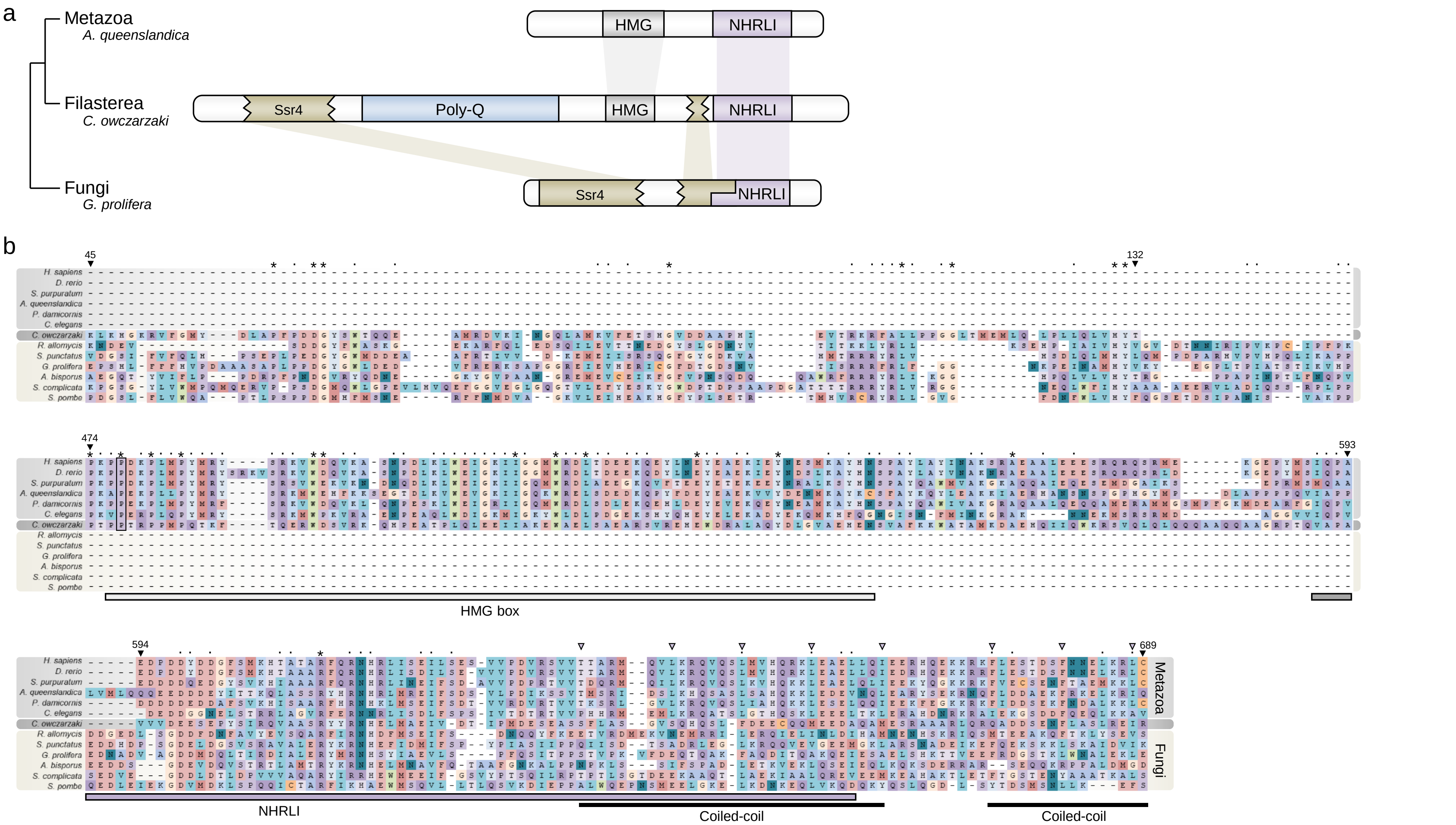

Supplementary Figure 2. Metazoan BAF57 and fungal Ssr4 proteins share a common origin. a) Domain architecture in different SMARCE proteins showing the relationship between the automatically detected HMG (High Mobility Group/HMG_box, PF00505), and Ssr4 (DUF1750/SWI-SNF_Ssr4, PF08549, IPR013859) domains, and the alignment-based detected NHRLI (unannotated) domain in representative opisthokonts. Poly-Q, poly-glutamine region in *Capsaspora owczarzaki* SMARCE. Right side cladogram indicates phylogenetic relationships between species. b) Multiple sequence alignment of the Ssr4 region (upper), HMG-containing region (middle), and NHRLI+KLCC region (lower) in SMARCE proteins of different species with lower boxes indicating specific features in the alignment. Numbers with arrows indicate the residue corresponding to *C. owczarzaki* SMARCE protein. The black-squared P477 residue is a signature of unspecific DNA-binding HMG domains[1]. Purple-filled arrows indicate hydrophobic residues of the heptad repeats in the coiled-coil region. Dots indicate highly conserved residues (same residue in 80% of the sequences), and asterisks deeply conserved residues. *H. sapiens*, *Homo sapiens*; *D. rerio*, *Danio rerio*; *S. purpuratum*, *Strongylocentrotus purpuratum*; *A. queenslandica*, *Amphimedon queenslandica*; *P. damicornis*, *Pocillopora damicornis*; *C. elegans*, *Caenorhabditis elegans*; *C. owczarzaki*, *Capsaspora owczarzaki*; *R. allomycis*, *Rozella allomycis*; *S. punctatus*, *Spizellomyces punctatus*; *G. prolifera*, *Gonapodya prolifera; A. bisporus*, *Agaricus bisporus*; *S. complicata*, *Saitoella complicata*; *S. pombe*, *Schizosaccharomyces pombe*.

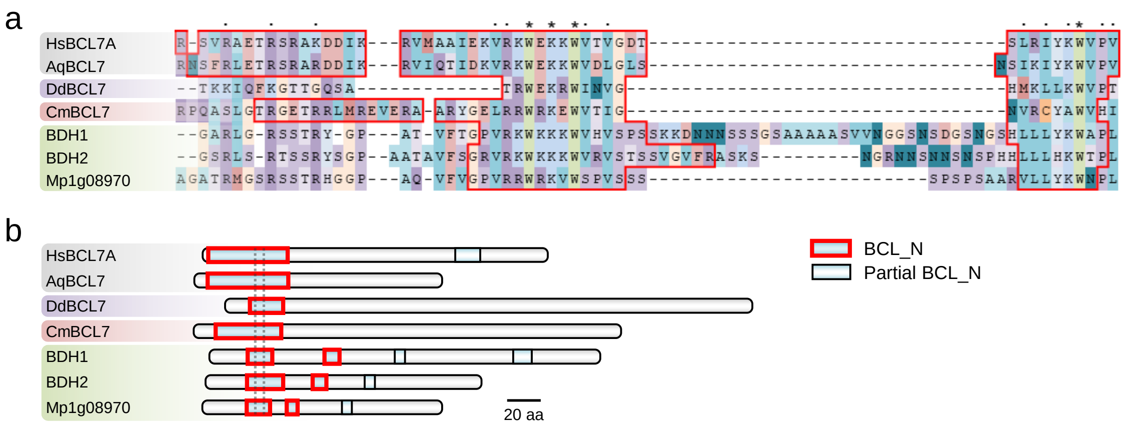

Supplementary Figure 3. SMARCJ proteins are present in animals, protozoans and plants. a) Multiple sequence alignment of BCL_N domains from different BCL7/SMARCJ proteins. Dots indicate highly conserved residues (same residue in 80% of the sequences), and asterisks deeply conserved residues. b) Representation of SMARCJ domain architectures as predicted by Pfam analysis. Partial BCL_N regions indicate non-significant hits of BCL_N related sequences. Doted lines narrow the position of the deeply conserved residues WxKxW. In a) and b), red-marked regions represent automatically predicted BCL_N (PF04714) regions. Grey shaded proteins are animal subunits; Purple shaded are amoebozoan subunits; red shaded are rhodophytan subunits; green shaded are plant subunits. HsBCL7A, *Homo sapiens* BCL7A; AqBCL7, *Amphimedon queenslandica* BCL7; DdBCL7, *Dictyostelium discoideum* BCL7; CmBCL7, *Cyanidioschizon merolae* BCL7.

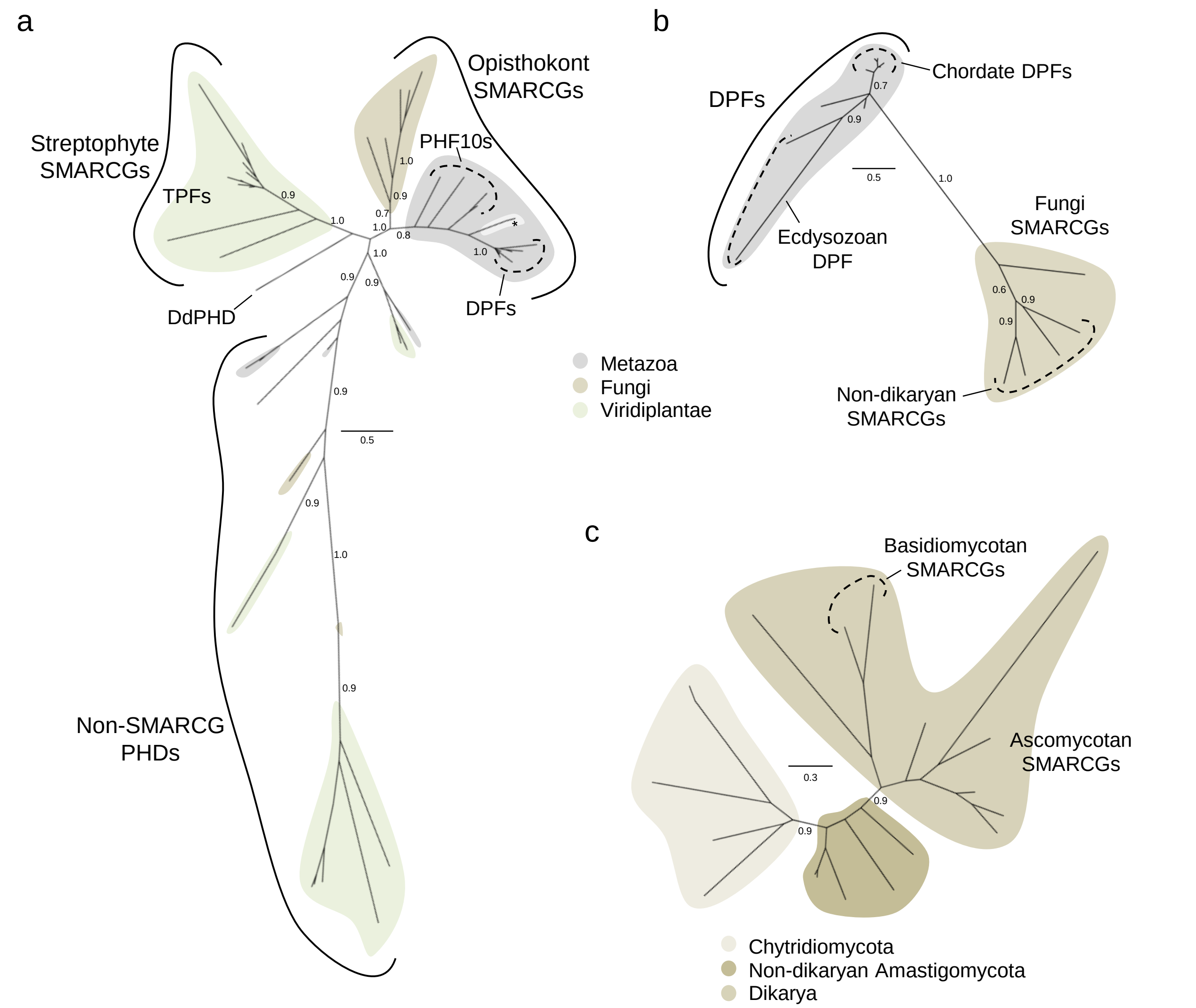

Supplementary Figure 4. SMARCG proteins contain multiple chromatin-related domains in different lineages. a) Maximum likelihood phylogenetic analysis of PHD domains from SMARCG proteins and related PHD-domains (Non-SMARCG PHDs, outgroup). b) Maximum likelihood phylogenetic analysis of Req domains (PF14051/IPR025750) from opisthokontan SMARCG proteins. c) Maximum likelihood phylogenetic analysis of CRC domains (CRC_subunit, PF08624) from fungal SMARCG proteins. Support values associated with branches represent maximum likelihood bootstrap values from 1000 replicates. Scale bar, ratio of substitutions per site. Raw phylogenetic tree files can be found at https://data.mendeley.com/datasets/6m4b8zrnpt/draft?a=d3801829-a6f1-4865-96b8-7d3138261e7b.

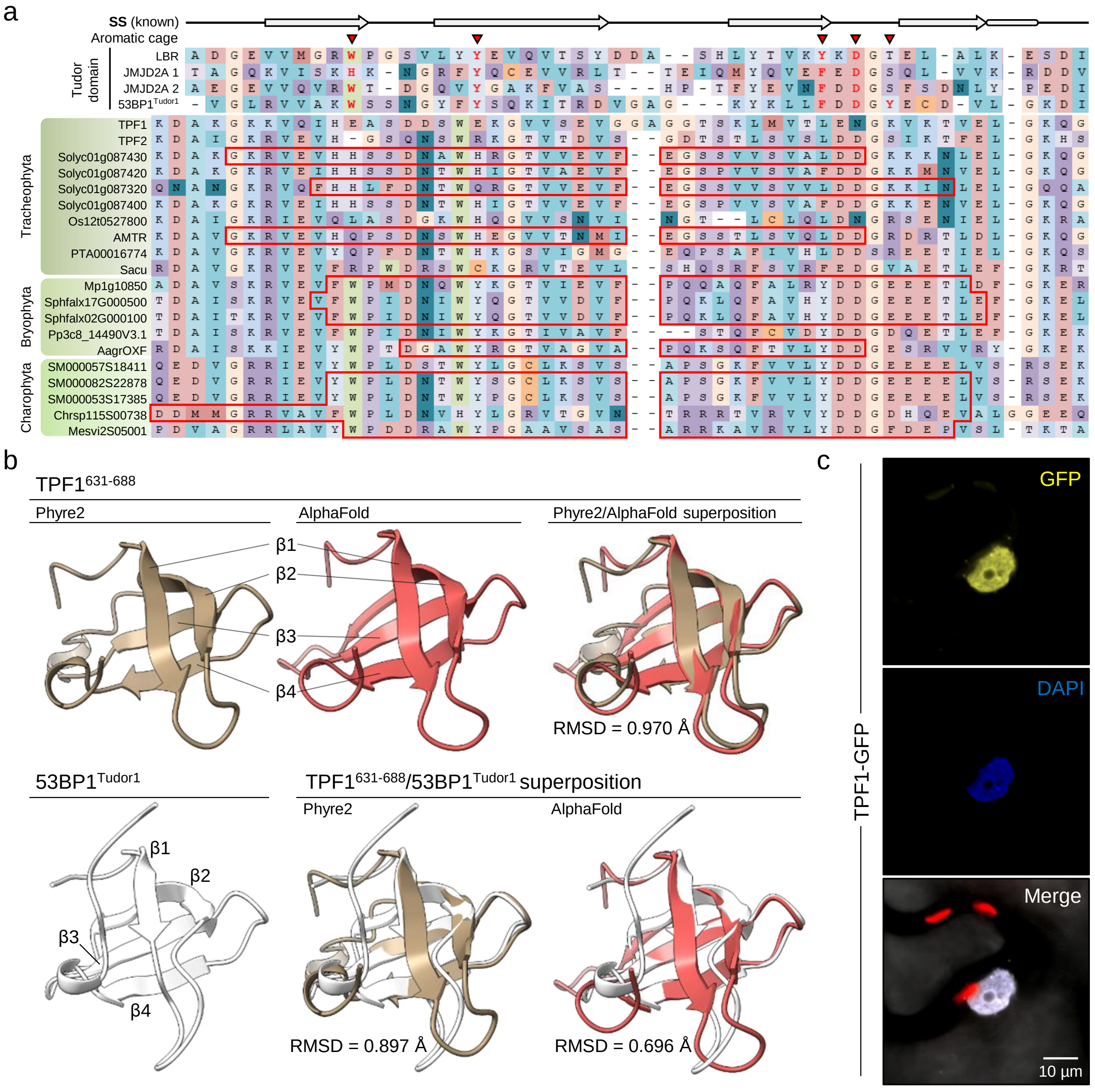

Supplementary Figure 5. TPF C-terminal region contains a conserved Tudor-like domain. a) Multiple sequence alignment of described Tudor domains (human LBR, JMJD2A, and 53BP1), and Tudor-like domains from multiple streptophytan TPF proteins. Known structure from human Tudor domains is depicted above (SS), in which right-pointing grey arrows represent β-sheets. Upper red arrows indicate known residues of the aromatic cage involved in specific binding to histone modifications[2]. Red residues are involved in either di- or trimethyllysine recognition. Red-marked regions represent automatically predicted Tudor-related regions (Agenet, PF05641; LBR_Tudor, PF09465; Tudor_3, PF18115). b) Ribbon structures of Phyre2- (brown) and AlphaFold- (red) predicted TPF1 Tudor-like domain and 53BP1 Tudor domain 1 (white). Structure superposition and RMSD calculation was obtained by structure-driven ChimeraX alignment. c) Confocal microscopy images of a TPF1-GFP fusion protein transiently expressed in *Nicotiana benthamiana* leaves. Yellow indicates GFP signal, blue, DAPI-stained nuclei, and red, chlorophyll autofluorescence. Upper panels show individual GFP and DAPI channels, and lower panel shiows merged GFP, DAPI, chlorophyll and bright light channels.

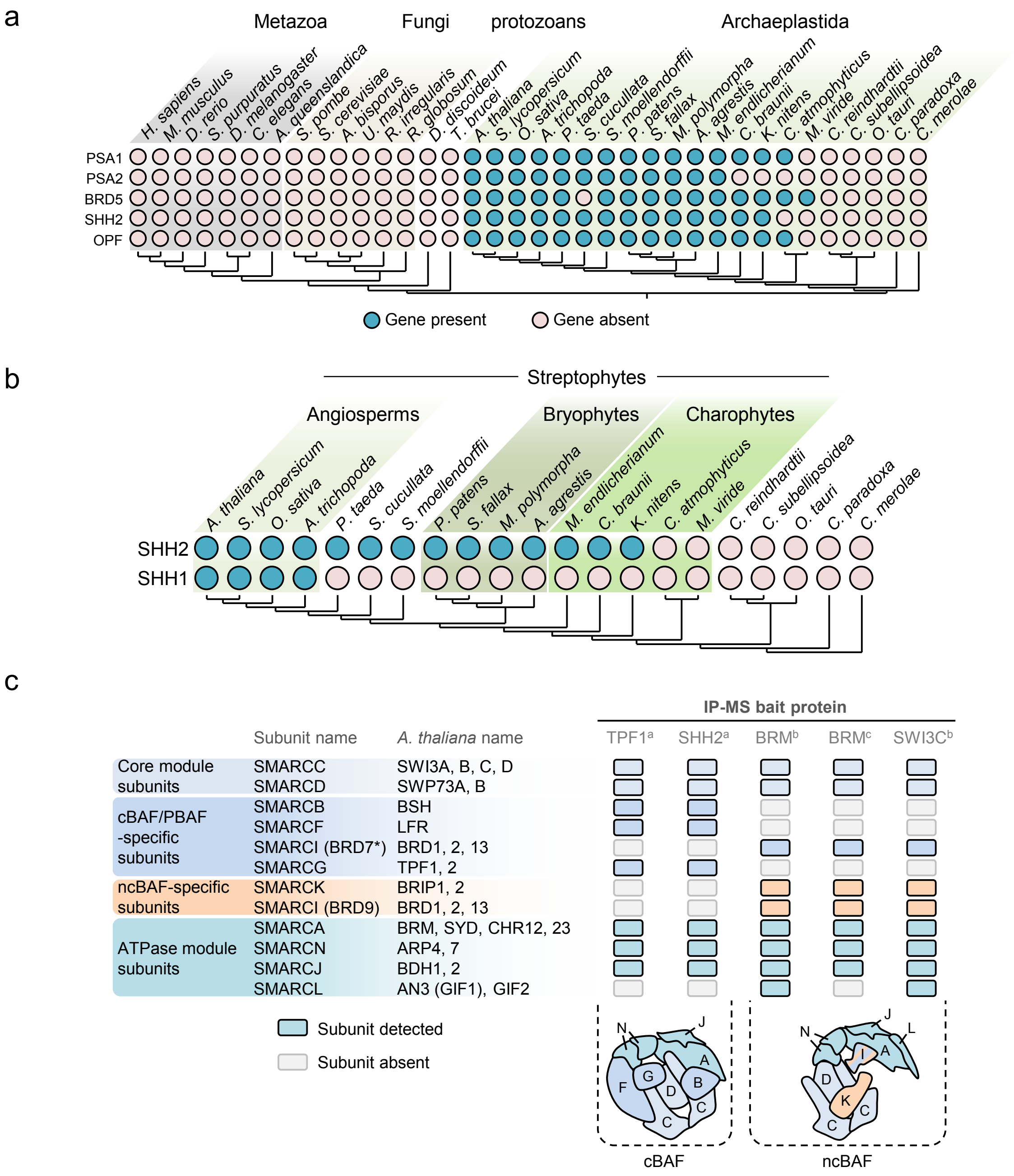

Supplementary Figure 6. Occurrence of SWI/SNF plant subunits. a) Occurrence of orthologs of the TPF1- and SHH2-complex uncharacterized subunits in eukaryotes. b) Occurrence of SHH1 and SHH2 paralogs in the Archaeplastida. Blue filled circles indicate at least a positive hit for the search of a given component (rows) in specific species (columns). Presence of a given homolog was determined as described in Materials and Methods. c) Occurrence of animal SWI/SNF subunits in plant SWI/SNF complexes. Animal subunits are divided into Core, BAF/PBAF-specific, ncBAF-specific, and ATPse modules[3]. Baits used for each plant IP-MS experiment are TPF1 and SHH2 (this study), BRM[4] and BRM and SWI3C[5]. Presence of ortholog subunits (rows) in specific IP-MS experiments (columns) is represented as filled squares of different colours associated to the four animal SWI/SNF modules. Absence of ortholog subunits is represented as a grey-filled square. A cartoon depicting the composition of the putative plant BAF and ncBAF complexes is shown. Plant-specific subunits are not depicted in these models.

Supplementary Table 1. Swi/Snf2 subunit searches summary

Subunit	Ancestry	Present	Absent	Present clades	Absent clades	Phmmer	Defining domains	Comments
SMARCA[†]	LECA	35	1	Eukaryotes	*T.b.* (distant ortholog)	No	Several domains.	*T. brucei* has other Snf2-like ATPases, associated to other complexes.
SMARCB	LECA	32	4	Eukaryotes	*T.b., P.p., C.s., O.t.*	No	Single SNF5 (PF04588) domain. Divided in two contiguous fragments outside fungi.	
SMARCC	LECA	35	1	Eukaryotes	*T.b.*	No	Myb_DNA-binding (PF00249), SWIRM (PF04433), SWIRM-assoc_1 (PF16495) / Metazoa and some fungi: SWIRM-assoc_2 (PF16496, also in D.d., U.m., R.i.) and SWIRM-assoc_3 (PF16498, also in R.i.) / Fungal Rsc8/Ssr2 and plant SWI3D: Zinc finger ZZ type (PF00569).	ZZ-type zinc finger-SMARCC exist in fungi and plants (SWI3D type), in many cases together with a zinc finger-less paralog. No phylogenetic relationship (independent loss or gain). TADA2α is the closest eukaryotic protein related to SMARCC.
SMARCD	LECA	35	1	Eukaryotes	*T.b.*	No	SWIB/MDM2 domain (PF02201), and absence of DEK_C (PF08766).	SMARCDs share ancestry with the UAF complex subunit Uaf30, DEK_C and SWIB domain containing proteins present in all eukaryotes but chordates and other specific species.
SMARCE	LOCA	10	26	Opisthokonts	*S.c.*, *U.m.*, *R.g.*, Archaeplastida	Yes	KLCC (unnanotated) / Filozoa: HMG_box (PF00505), BAF57 (IPR030089) / Fungi and Filasterea: SWI-SNF_Ssr4 (PF08549, IPR013859)	Clear relationship between metazoan BAF57 and fungal Ssr4 with the filasterean *C. owczarzaki* representing an intermediate state protein with all the domains. Lost in several fungi. Snf6 in yeast fulfills a similar role, but no phylogenetic relationship. Filozoan HMG is similar to those of NHP6 and SSRP1 in eukaryotes.
SMARCF	LECA	30	1/5	Eukaryotes	*T.b.* (ortholog in close species)/*D.d.*, *M.v.*, *C.r.*, *O.t.*, *C.p.*	Yes	ARID1/Swi1: ARID (PF01388), BAF250_C (PF12031)/ ARID2/Rsc9: ARID (PF01388), RFX_DNA_Binding (PF02257)/ ARM-type_fold (IPR016024), Armadillo (IPR00025). Additional domains: Znf_C2H2_type (IPR013087) in metazoan ARID2.	See Figure 2 and Supplementary Figure 1. While *D. discoideum* does not seem to harbour SMARCF proteins, the closely related species *A. castellanii* does.
SMARCG	LECA	29	7	Eukaryotes	*T.b.*, *C.b.*, Chlorophyta, Glaucophyta, Rhodophyta	Yes	Several domains.	See Figure 3 and Supplementary Figure 4.
SMARCH[†]	LOCA	13	23	Opisthokonts	*D.d.*, *T.b.*, Archaeplastida	Yes	Multiple bromodomain (PF00439), multiple or single Bromo-adjacent homology (BAH, PF01426)/ metazoa PBRM1-type : HMG_box (PF00505).	BRD7/9 and PBRM1 are unambiguously found together as different proteins in non-ascomycotan fungi, while bromodomain from both types of proteins give Rsc1, Rsc2, Rsc4 as hits in Ascomycota. See†. PBRM1 architectures (4xBRD+1-2xBAH), but with no HMG box can be found outside metazoa in other Holozoa groups (i.e.: Ichthyosporea, Filasterea, Choanoflagellatea). Evolutionary pathway of SMARCH by either gene fusión (of several Rsc1,2,4-like architecture to form a PBRM1-like) or gene split (of a PBRM1-like into several Rsc1,2,4-like) remains unsolved.
SMARCI[†]	LECA	32	2/2	Eukaryotes	Ascomycota/*R.g.*, *C.p.* (ortholog in close species)	Yes	Bromodomain (PF00439) / Metazoa: DUF3512 (PF12024) / Non-angiosperm streptophytes: PEHE domain (PF15275).	BRD7/9 duplication seems to be unique to chordates. In some plants these proteins also contain PEHE domains (mostly bryophytes and algae), a domain linked to histone marking.
SMARCJ	LECA	25	11	Eukaryotes	Fungi, T.b., Chlorophyta, Glaucophyta	Yes	BCL_N (PF04714) domain.	PF04714 cannot be found in fungi using any available data source.
SMARCK	LECA	30	6	Eukaryotes	*A.q.*, Ascomycota, *R.g.*, *T.b.*, *C.a.*	Yes	GLTSCR1 (PF15249) domain.	PF15249 domains are absent in virtually all available Ascomycota and Kinetoplastids.
SMARCL	LECA	26	10	Eukaryotes	Dikarya, *R.g.*, *D.d.*, *T.b.*, *C.b.*, *M.v.*, *C.r.*	Yes		Unambiguosly found in fungi and linked to *S.p.* Ssr complex Snf30 subunit. *Rhizoclosmatium* may contain a divergent Snf30 with no SSXT domain.
SMARCM	LMCA	6	30	Metazoa (Nephrozoa[a])	*A.q.*, Fungi, Archaeplastida	Yes	Multiple zf-C2H2 (PF00096) domains.	BCL11A/B gene duplication seems to be found in chordates. Ecdysozoans and *S. purpuratus* have a single copy BCL11. Extended search outside Eumetazoa rendered only distant C2H2-related genes from bacteria.
SMARCN	LECA	36	0	Eukaryotes	N/A	No	Single Actin (PF00022) domain.	
Snf6	Class-specific	1	35	Saccharomycetales (Class, several species), Lactobacillales (Class, *Abiotrophia* sp.)	Most	Yes	Distant AGO_PAZ (PF18309)-related domain.	Distant hits are uncultured Archaea Rieske-domain proteins. Kiwi fruit (*Actinidia*) has a hit 100% identical to *S. cerevisiae* Snf6 considered as a contamination.
Rtt102	Class-specific	1	35	Saccharomycetales (Class, several species)	Most	Yes	Single Rtt102p domain (PF09510) protein.	
Rsc3/Rsc30	Dikarya	2	2/32	*S.c., U.m.*	*S.p.*, *A.b.* (distant ortholog)/*D.d.*, *T.b.*, Metazoa, Archaeplastida	Yes	Gal4-dimer (PF03902) or Fungal-specific transcription factor domain (PF04082) containing protein.	
Rsc58	Saccharomycetales	2	34	*S.c.*, *S.p.*, other ascomycetes, specially saccharomycetales	Most	Yes	Partial BRD domain.	*S.p.* and *S.c.* Rsc58 proteins are highly divergent and neither can be found using the other.
Lbd7/Rsc14	Saccharomycetales	1	35	*S.c.*	Most	Yes	Rsc14 (PF08586).	
Htl1	Saccharomycetales	1	35	*S.c.*	Most	Yes	No domain.	Fulfills a similar structural role in RSC as SMARCE1 in BAF complexes, but no phylogenetic relationship exists.
Taf14	Fungi	3	33	*S.c.*, *S.p.*, *R.i.*	*R.g.*, *A.b.*,*U.m.*, *D.d.*, *T.b.*, Metazoa, Archaeplastida	Yes	YEATS (PF03366).	Distantly related YEATS-containing YAF9/AF9 proteins are conserved in all lineages. Some YAF9 proteins in plants are incorrectly annotated as TAF14 proteins.
Snf11	Saccharomycetales	1	35	*S.c.*	Most	Yes	No domain.	
PSA1	Streptophyta	15	21	Streptophyta (with exceptions)	*M.v.*, Metazoa, Fungi, Chlorophyta, Rhodophyta, Glaucophyta	Yes	DUF702 (PF05142).	
PSA2	Streptophyta	12	24	Embryophytes, *Sp. m.* (plus other zygnematalean algae)	Metazoa, Fungi, Charopyta (with exceptions), Chlorophyta, Rhodophyta, Glaucophyta	Yes	Some cases: RWP-RK (PF02042).	Clear orthologs are found within land plants. Zygnematalen sequences are also good hits. Other hits are RWP-RK transcription factors.
SHH2	Streptophyta	14	22	Streptophyta (with exceptions)	*C.a.*, *M.v.*, Metazoa, Fungi, Chlorophyta, Rhodophyta, Glaucophyta	Yes	SAWADEE (PF16719), SAWADEE_dom (IPR032001), Homeobox-like_sf (IPR009057), Homeobox_dom (IPR001356).	

A.b., *Agaricus bisporus*; *A. q.*, *Amphimedon queenslandica*; *C.a.*, *Chlorokyus atmophyticus*; *C.b.*, *Chara braunii*; *C.p.*, *Cyanophora paradoxa*; *C.r.*, *Chlamydomonas reindhardtii*; *C.s.*, *Coccomyxa subellipsoidea*; *D.d.*, *Dictyostelium discoideum*; *M.v.*, *Mesostigma viride*; *O.t.*, *Ostreococcus tauri*; *P.p.*, *Physcomitrium patens*; *R.i.*, *Rhizophagus irregularis*; *R.g.*, *Rhizoclosmatium globosum*; *S.c.*, *Saccharomyces cerevisiae*; *S.p.*, *Schizosaccharomyces pombe*; *Sp.m.*, *Spirogloea muscicola*; *T.b.*, *Trypanosomas brucei*; *U.m.*, *Ustilago maydis*.

[a]SMARCM/BCL11 might be present in choanozoans, filozoans or holozoans. See Comments.

[†]Bromodomains of different proteins within certain species are more related between each other than among orthologs. This is specially seen in yeasts due to gene conversion mechanisms.

Supplementary Table 2. Species and databases used in this study.

Kingdom	Sub-clade	Species	Genome	Database	Ref.
Metazoa	Chordata	*Homo sapiens*	all versions	NCBI	
		Mus musculus	all versions	NCBI	
		Danio rerio	all versions	NCBI	
	Echinodermata	*Strongylocentrotus purpuratus*	all versions	NCBI	
	Arthropoda	*Drosophila melanogaster*	all versions	NCBI	
	Nematoda	*Caenorhabditis elegans*	all versions	NCBI	
	Porifera	*Amphimedon queenslandica*	all versions	NCBI	
Archeaplastida	Streptophyta	*Arabidopsis thaliana*	TAIR11	TAIR	
		Solanum lycopersicum	iTAG4.1	SolGenomics	
		Oryza sativa	IRGSP 1.0 protein	Rice Genome Annotation Project	6
		Amborella trichopoda	AMTR 1.0 prot	Phytozome/Proteome	
		Pinus taeda/Picea abies	2.0/1.0 assemblies	Congenie	
		Salvinia cucullata/Azolla filiculoides	Genome v1.2	Fernbase	7
		Selaginella moellendorffii	v1.0	Phytozome	
		Anthoceros agrestis	Bonn and Oxford strains	www.hornworts.uzh.ch	8
		Physcomitrella patens	V3.3	Phytozome	
		Sphagnum fallax	Genome v1.1	Phytozome	
		Marchantia polymorpha	Tak-1 genome v5.1	marchantia.info	
		Mesotaenium endlicherianum	SAG 12.97 genome	PhycoCosm	9
		Chara braunii	S276 genome	ORCAE/PhycoCosm	10
		Klebsormidium nitens	NIES-2285 genome v1.1	PhycoCosm	11
		Chlorokybus atmophyticus	CCAC 0220	CNGB/PhycoCosm	12
		Mesostigma viride	CCAC 1140 genome	CNGB/PhycoCosm	12
	Chlorophyta	*Chlamydomonas reindhardtii*	genome v5.6	Phytozome/PhycoCosm	13
		Coccomyxa subellipsoidea	C-169	Phytozome/PhycoCosm	14
		Ostreococcus tauri	RCC1115 genome v1.0	Phytozome/PhycoCosm	15
	Rhodophyta	*Cyanodioschyzon merolae*	Soos genome	PhycoCosm	16
	Glaucophyta	*Cyanophora paradoxa*	CCMP329 genome	cyanophora.rutgers.edu	17
Fungi	Ascomycota	*Schizosaccharomyces pombe*		NCBI	
		Saccharomyces cerevisiae		NCBI	
	Basidiomicota	*Agaricus bisporus*		MycoCosm	
		Ustilago maydis		NCBI	
	Mucoromycota	*Rhizophagus irregularis*		MycoCosm	
	Chytridiomycota	*Rhizoclosmatium globosum*		NCBI	
Protozoans	Amoebozoa	*Dictyostelium discoideum*		DictyBase/NCBI	
	Euglenozoa	*Trypanosoma brucei*		EnsemblProtist	

OneKP and PhycoCosm database was used to confirm poorly annotated plant genomes
EnsemblProtist and NCBI for additional kinetoplastid and protozoos
Mycocosm and NCBI for additional fungal genomes

Supplementary References

1. O'Flaherty, E. & Kaye, J. TOX defines a conserved subfamily of HMG-box proteins. BMC Genomics 4, 13, doi:10.1186/1471-2164-4-13 (2003).
2. Tong, Q. et al. Structural Plasticity of Methyllysine Recognition by the Tandem Tudor Domain of 53BP1. Structure 23 (2), 312-321,doi:10.1016/j.str.2014.11.013 (2015).
3. Mashtalir, N. et al. Modular Organization and Assembly of SWI/SNF Family Chromatin Remodeling Complexes. Cell 175, 1272-1288.e1220, doi:10.1016/j.cell.2018.09.032 (2018).
4. Jarończyk, K. et al. Bromodomain-containing subunits BRD1, BRD2, and BRD13 are required for proper functioning of SWI/SNF complexes in Arabidopsis. Plant communications 2, 100174, doi:10.1016/j.xplc.2021.100174 (2021).
5. Yu, Y. et al. BRAHMA-interacting proteins BRIP1 and BRIP2 are core subunits of Arabidopsis SWI/SNF complexes. Nat Plants 6, 996-1007, doi:10.1038/s41477-020-0734-z (2020).
6. Kawahara, Y. et al. Improvement of the Oryza sativa Nipponbare reference genome using next generation sequence and optical map data. Rice 6 (1), 4, doi: 10.1186/1939-8433-6-4 (2013).
7. Li, FW. et al. Fern genomes elucidate land plant evolution and cyanobacterial symbioses. Nature Plants 4 (7), 460-472, doi:10.1038/s41477-018-0188-8 (2018).
8. Li, FW. et al. Anthoceros genomes illuminate the origin of land plants and the unique biology of hornworts. Nature Plants 6 (3), 259-272, doi: 10.1038/s41477-020-0618-2 (2020).
9. Cheng, S. et al. Genomes of Subaerial Zygnematophyceae Provide Insights into Land Plant Evolution. Cell 179 (5), 1057-1067.e14, doi:10.1016/j.cell.2019.10.019 (2019).
10. Nishiyama, T. et al. The Chara Genome: Secondary Complexity and Implications for Plant Terrestrialization. Cell 174 (2), 448-464.e24, doi:10.1016/j.cell.2018.06.033 (2018).
11. Hori, K. et al. Klebsormidium flaccidum genome reveals primary factors for plant terrestrial adaptation. Nature Communications 5, 3978, doi:10.1038/ncomms4978 (2014).
12. Wang, S. et al. Genomes of early-diverging streptophyte algae shed light on plant terrestrialization. Nature Plants 6(2):95-106. doi: 10.1038/s41477-019-0560-3 (2020).
13. Merchant, S.S. et al. The Chlamydomonas genome reveals the evolution of key animal and plant functions. Science 318 (5848), 245-250, doi:10.1126/science.1143609 (2007).
14. Blanc, G. et al. The genome of the polar eukaryotic microalga Coccomyxa subellipsoidea reveals traits of cold adaptation. Genome Biology 13 (5), R39, doi:10.1186/gb-2012-13-5-r39 (2012).
15. Blanc-Mathieu, R. et al. Population genomics of picophytoplankton unveils novel chromosome hypervariability. Science Advances 3 (7), e1700239, doi:10.1126/sciadv.1700239 (2017).
16. Rossoni, A.W. et al. The genomes of polyextremophilic cyanidiales contain 1% horizontally transferred genes with diverse adaptive functions. Elife 8:e45017, doi:10.7554/eLife.45017 (2019).
17. Price, D.C. et al. Analysis of an improved Cyanophora paradoxa genome assembly. DNA Research 26 (4), 287-299, doi:10.1093/dnares/dsz009 (2019).
