# Supplementary material for: Comprehensive identification of SWI/SNF complex subunits underpins deep eukaryotic ancestry and reveals new plant components
Source: Commun Biol. 2022 Jun 6;5:549. doi: 10.1038/s42003-022-03490-x (PMC9170682; doi:10.1038/s42003-022-03490-x)
Supplement: Supplementary file 3 — Description of Additional Supplementary Files [file 42003_2022_3490_MOESM3_ESM.pdf]

Description of Additional Supplementary Files

File name: Supplementary Data 1
Description: Mass spectrometry raw data and analyses related to Table 2
